# Torquetenovirus in pregnancy: Correlation with vaginal microbiome, metabolome and pro-inflammatory cytokines

**DOI:** 10.3389/fmicb.2022.998849

**Published:** 2022-09-09

**Authors:** Sara Morselli, Claudio Foschi, Luca Laghi, Sara Zagonari, Giulia Patuelli, Tania Camboni, Camilla Ceccarani, Clarissa Consolandi, Marielle Ezekielle Djusse, Maria Federica Pedna, Antonella Marangoni, Marco Severgnini, Vittorio Sambri

**Affiliations:** ^1^Microbiology Unit, Department of Specialized, Experimental and Diagnostic Medicine (DIMES), University of Bologna, Bologna, Italy; ^2^Microbiology Unit, IRCCS Azienda Ospedaliero-Universitaria di Bologna, Bologna, Italy; ^3^Department of Agricultural and Food Sciences (DISTAL), Centre of Foodomics, University of Bologna, Cesena, Italy; ^4^Interdepartmental Centre for Agri-Food Industrial Research (CIRI Agrifood), University of Bologna, Cesena, Italy; ^5^Family Advisory Health Centres, Ravenna, Italy; ^6^Institute of Biomedical Technologies, National Research Council, Segrate, Italy; ^7^Great Romagna Hub Laboratory, Unit of Microbiology, Pievesestina di Cesena, Italy

**Keywords:** torquetenovirus, TTV, vaginal microbiome, pregnancy, vaginal metabolome, women's health

## Abstract

Torquetenovirus (TTV) is a negative sense, single-stranded DNA virus present in many body fluids of apparently healthy individuals. At present, it is considered a non-pathogenic endogenous virus. TTV can be detected in the vagina of pregnant women, its abundance being modulated with the extent of immune system activation. Until now, there is only scarce information regarding the association between TTV and the composition of the vaginal environment. Therefore, this study aimed to assess the presence of TTV in the vaginal ecosystem of a cohort of white women with a normal pregnancy (*n* = 60) at different gestational stages (first, second and third trimester) and in 9 subjects suffering a first trimester miscarriage. For each woman, we determined (i) the presence and titer of TTV, (ii) the vaginal bacterial composition by means of Nugent score and 16S rRNA gene sequencing, (iii) the vaginal metabolic profiles through ^1^H-NMR spectroscopy, and (iv) the vaginal concentration of two pro-inflammatory cytokines (IL-6 and IL-8). More than one third of women were found negative for TTV at all gestational stages. Although not statistically significant, the positivity for TTV dropped from 53.3% in the first to 36.6% in the third trimester. TTV loads varied greatly among vaginal samples, ranging between 2 × 10^1^ and 2 × 10^5^ copies/reaction. No difference in TTV prevalence and loads was observed between women with normal pregnancies and miscarriages. The presence of TTV was more common in women with a higher vaginal leucocyte count (*p* = 0.02). The levels of IL-6 (*p* = 0.02), IL-8 (*p* = 0.03), propionate (*p* = 0.001) and cadaverine (*p* = 0.006) were significantly higher in TTV-positive samples. TTV titer was positively correlated with the concentrations of 4-hydroxyphenyllactate (*p* < 0.0001), isoleucine (*p* = 0.01) and phenylalanine (*p* = 0.04). TTV-positive samples were characterized by a higher relative abundance of *Sneathia* (*p* = 0.04) and *Shuttleworthia* (*p* = 0.0009). In addition, a trend toward a decrease of *Lactobacillus crispatus* and *Lactobacillus jensenii*, and an increase of *Lactobacillus iners* was observed for TTV-positive samples. In conclusion, we found that TTV is quite common in women with normal pregnancy outcomes, representing a possible predictor of local immune status.

## Introduction

Throughout a woman's lifespan, the vaginal microbiome undergoes major changes in response to various factors, such as hormonal levels, sexual habits, hygiene, pregnancy, pharmaceutical treatments, and urogenital infections (Kroon et al., 2018; Parolin et al., 2018; Ceccarani et al., 2019; Severgnini et al., 2022).

During healthy pregnancies, the vaginal microbiome is usually characterized by a significant decrease in overall bacterial diversity, an increased stability, and an enrichment of *Lactobacillus* spp. (Dall'Asta et al., 2021; Marangoni et al., 2021). A lactobacilli-dominated vaginal microbiota is associated with low inflammation and low immune system activation, thus contributing to the maintenance of maternal-fetal health (Witkin et al., 2019).

In the case of bacterial vaginosis (BV), a condition of vaginal dysbiosis characterized by a depletion of lactobacilli and an overgrowth of several anaerobes (e.g., *Gardnerella vaginalis, Fannyhessea vaginae, Prevotella* spp., *Megasphaera* spp.), an increased local inflammation is present, with the risk of pregnancy-related complications and preterm birth (Prince et al., 2014; Anahtar et al., 2015; Di Simone et al., 2020).

The changes in the vaginal bacterial communities are accompanied by profound alterations in the composition of vaginal metabolites. High concentrations of biogenic amines (e.g., putrescine, cadaverine, and trimethylamine) and short-chain fatty acids (SCFAs) are the most common fingerprints of BV (Srinivasan et al., 2015; Parolin et al., 2018).

Recently, it has been shown that Torquetenovirus (TTV), a non-pathogenic endogenous virus, can be detected in the vagina of pregnant women, its abundance being modulated with the extent of immune system activation (Maggi and Bendinelli, 2010; Tozetto-Mendoza et al., 2020, 2022). TTV has a worldwide distribution, and it can be transmitted by multiple routes, including bloodborne, oro-fecal, respiratory, and sexual transmission (Maggi and Bendinelli, 2010; Haloschan et al., 2014). This virus appears to replicate mainly in T lymphocytes, but the exact cellular receptors for TTV are still unknown. Anyway, even though TTV can be considered an orphan virus, TTV viremia may potentially be a simple and sensitive measure of immune system function of the host (Shibayama et al., 2001; Maggi et al., 2010).

Until now, there is only scarce information on the association between TTV titer and the microbial/metabolic composition of the vaginal environment (Tozetto-Mendoza et al., 2020, 2022). Moreover, the mechanisms associated with variations in vaginal TTV titer, as well as the relevance of monitoring TTV loads in pregnancy remain open questions.

Therefore, in this study we assessed the presence of TTV in the vaginal ecosystem of a cohort of white women with a normal pregnancy (*n* = 60) at different gestational stages (i.e., first, second and third trimester) and in 9 subjects suffering a first trimester miscarriage. For each woman, we determined (i) the presence and titer of TTV in the vaginal ecosystem by means of a real-time PCR assay, (ii) the vaginal bacterial composition by means of a microscopic scoring system (Nugent score) and by sequencing of the V3–V4 hypervariable regions of the 16S rRNA gene, (iii) the vaginal metabolic profiles through ^1^H-NMR spectroscopy, and (iv) the vaginal concentration of two pro-inflammatory cytokines (IL-6 and IL-8).

## Materials and methods

### Study group and sample collection

From April 2019 all the white pregnant women attending the Family Advisory Health Centers of Ravenna (Italy) were considered eligible for the study. Exclusion criteria included the following: (i) age<18 years; (ii) being positive for HIV infection; (iii) obesity (body mass index >33); (iv) medically assisted procreation; (v) use of antimicrobials in the month prior the enrollment; (vi) use of vaginal topical agents in the 2 weeks before the enrollment; (vii) presence of chronic diseases (e.g., diabetes, autoimmune disorders, malignancies); (viii) drug addiction or heavy smokers (>15 cigarettes/day). Furthermore, women were excluded if a diagnosis of sexually transmitted infections (STIs) (i.e., *Chlamydia trachomatis, Neisseria gonorrhoeae, Trichomonas vaginalis, Mycoplasma genitalium*) or aerobic vaginitis was made. For each woman a clinical visit was performed at gestational stages 9–13 weeks (first trimester), 20–24 weeks (second trimester), and 32–34 weeks (third trimester). At each time point, two vaginal swabs were collected: the first one (E-swab, Copan, Brescia, Italy) was used for microbiological tests, while the second was collected with a sterile cotton bud, re-suspended in 1 ml of sterile saline, and stored at−80°C until use. Frozen vaginal swabs were thawed, vortexed for 1 min and removed from the liquid. After centrifugation (10,000 × g for 15 min) cell-free supernatants were used for metabolomic analysis and cytokine detection, whereas cell-pellets were employed both for TTV detection and vaginal microbiome profiling (see specific paragraphs).

### Ethics statement

The study protocol was approved by the Ethics Committee of Romagna (CEROM) (no 2032 of 21st February 2018) and it was carried out in accordance with the Declaration of Helsinki. Each woman gave written informed consent to participate in the study.

### Microbiological investigations

A commercial nucleic acid amplification technique (NAAT) was used to exclude the presence of STIs (Seeplex STI Master Panel 1, Seegene, Seoul, South Korea), whereas candidiasis and aerobic vaginitis diagnosis was performed by microscopic examination and microbial cultures, as described elsewhere (Donders et al., 2011; Yano et al., 2019).

Based on Nugent score, a Gram stain scoring system evaluating for the presence of different bacterial morphotypes (Nugent et al., 1991), women were categorized into 3 groups: ‘H' (healthy; score 0–3; normal lactobacilli-dominated microbiota), ‘I' (score 4–6; intermediate microbiota), ‘BV' (score 7–10; bacterial vaginosis) (Zozaya-Hinchliffe et al., 2010).

The presence of vaginal leukocytes (white blood cells: WBCs) was evaluated after visualization of a minimum of five fields under light microscopy at 400×. Samples were categorized as ‘minimal or no inflammation' in case of<5 WBCs in all visualized fields or as ‘significant inflammation' in presence of ≥5 WBCs in at least one field visualized (Geisler et al., 2004).

### Vaginal microbiome profiling

Nucleic acids were extracted from vaginal swabs by means of the Versant molecular system (Siemens Healthcare Diagnostics, Tarrytown, NY, USA) (Marangoni et al., 2015). Afterwards, the V3–V4 hypervariable regions of the bacterial 16S rRNA gene were amplified according to the 16S metagenomic sequencing library preparation protocol (Illumina, San Diego, CA, USA), as previously described (Severgnini et al., 2021). Raw reads were analyzed according to the procedure reported by (Severgnini et al., 2021).

Zero-radius Operational Taxonomic Units (zOTUs) creation, taxonomy assignments, and diversity analyses were performed using the QIIME suite (release 1.9.0) (Caporaso et al., 2010), unoise3 algorithm (Edgar, 2016), RDP classifier (Wang et al., 2007), and SILVA 16S rRNA database (release 132, https://www.arb/silva.de/fileadmin/silva_databases/qiime/Silva_132_release.zip).

As already reported, characterization of *Lactobacillus* spp. was performed by BLAST-aligning all reads belonging to that genus to a custom reference database (Ceccarani et al., 2019).

Alpha-diversity was evaluated according to several microbial diversity metrics (i.e., chao1, Shannon index, observed species, Good's coverage, and Faith's phylogenetic distance), whereas beta-diversity analysis was performed using both weighted and unweighted Unifrac metrics (Lozupone et al., 2011), and through the Principal Coordinates Analysis (PCoA).

### Detection of TTV in the vaginal ecosystem

Starting from the remaining DNA eluate, all the vaginal swabs were tested for the presence of TTV as previously reported (Maggi et al., 2001; Tozetto-Mendoza et al., 2022).

The PCR reaction mixtures (final volume: 25 μL) included 12.5 μL of Platinum Quantitative PCR Supermix-UDG with ROX (Invitrogen, Waltham, MA, USA), 250 nM of primers, 62 nM of the probe, and 2.5 μL of template. All PCR reactions were performed with the following cycling conditions using a QuantStudio Real-Time PCR system (Applied Biosystems, Waltham, MA, USA): 2 min at 50°C, 15 s at 95°C, and 40 cycles of 15 s at 95°C and 60 s at 60°C.

A standard curve with known amounts of a synthetic oligonucleotide was used for TTV quantification (Tozetto-Mendoza et al., 2022). Results were expressed as log10 DNA copies/reaction.

### Cytokine detection

The concentration of IL-6 (pg/ml) and IL-8 (pg/ml) was determined on the cell-free supernatants of the vaginal swabs by means of commercial ELISA assays (Simple Plex Human IL-6 and IL-8 Cartridges, R&D Systems, Minneapolis, MN, USA), following manufacturer's instructions (Brys et al., 2020).

### Metabolomic analysis

Metabolomic analysis was performed by means of a ^1^H-NMR spectroscopy starting from 700 μl of the cell-free supernatants of the vaginal swabs, using an AVANCE III spectrometer (Bruker, Milan, Italy), as previously reported (Foschi et al., 2018; Zhu et al., 2019). The signals were assigned by comparing their multiplicity and chemical shift with Chenomx software data bank (ver 8.3, Chenomx Inc., Edmonton, Alberta, Canada).

### Data analysis and statistics

Statistical analyses were conducted by using GraphPad Prism software (version 5.02; GraphPad Software, San Diego, CA, USA) and Matlab (Software version 7.7.0, Natick, MA, USA). Fisher's exact test was used to compare categorical data (i.e., presence of TTV stratified by the vaginal status), whereas ANOVA test, followed by Tukey's multiple comparisons test, was employed to compare TTV loads among the different categories. TTV loads were correlated to metabolite concentrations by calculating the Spearman correlation coefficient.

Statistical evaluation of the alpha-diversity indices was performed by non-parametric Monte Carlo-based tests, whereas beta-diversity differences were assessed by a permutation test with pseudo F-ratios (“adonis” function from R package “vegan”, version 2.0-10 Oksanen et al., 2013). Pairwise relative abundance analysis was performed using a non-parametric Mann–Whitney U test.

Statistical significance (*p*-value <0.05) was assessed after adjustment for multiple comparisons (i.e., Benjamini-Hochberg correction, with a FDR of 0.25).

Correlation between microbial composition at the genus level and presence/absence of TTV was calculated using the point biserial correlation (Gupta, 1960), whereas the correlation between microbial profiles and TTV loads (log-transformed TTV copy number) was performed using Spearman's rank-based correlation coefficient. Only coefficients showing a *p*-value of the linear model<0.05 were considered.

### Data availability

Raw sequencing data of 16S rRNA gene are available at NCBI Short-reads Archive (SRA) with BioProject accession number PRJNA766806 (https://www.ncbi.nlm.nih.gov/sra/PRJNA766806).

## Results

### Study population

A total of 60 pregnant women with a median age of 31 years (min–max: 21–44) completed the study. In addition, 9 women (median age: 35 years; min–max: 23–41) who had a spontaneous first trimester miscarriage (gestational age: 11–13 weeks) were also included.

Overall, excluding specimens from women with miscarriages, 118 vaginal samples (65.6%) were characterized by a lactobacilli-dominated flora (Nugent score 0–3), 43 (23.9%) by an intermediate microbiota (Nugent score: 4–6), and the remaining 19 (10.5%) harbored a BV-associated bacterial composition (Nugent score: 7–10).

It is noteworthy that a significant reduction of dysbiotic cases was noticed (*p* = 0.002) when moving from the first to the third trimester of pregnancy. Finally, women who suffered a first trimester miscarriage (*n* = 9) were mainly characterized by a condition of dysbiosis (i.e., 6 with an intermediate microbiota and 2 with a BV condition).

### Detection of TTV

Overall, considering all the specimens belonging to the 60 women who completed the study, 42.7% (77/180) of the tested vaginal swabs were positive for TTV.

Stratifying the samples by the gestational age (*n* = 60 per time point), there was a non-significant decrease in TTV positivity between samples obtained in the first, second and third trimester. In fact, 32 (53.3%) were TTV-positive in the first trimester, 23 (38.3%) in the second, and 22 (36.6%) in the third. No difference in TTV prevalence was found when comparing normal pregnancies with miscarriages: in fact, TTV was detected in about half (5/9; 55.5%) of the women who suffered a first trimester miscarriage.

Considering each subject throughout the pregnancy, more than one third of women were found negative for TTV at all three trimesters of pregnancy (25/60; 41.6%). Conversely, 26.6% of women (16/60) were positive for TTV at each trimester. Thirteen of the remaining cases were characterized by a TTV positivity in the first and/or second trimester, with a negativity at the end of pregnancy.

TTV loads (expressed as log10 DNA copies/reaction) varied greatly among vaginal samples, ranging between 1.4 (about 26 copies/reaction) and 5.3 (about 209,000 copies), with a mean (± standard deviation, SD) of 3.06 ± 0.96. No significant difference in TTV titer was found between women with a miscarriage and women with a normal pregnancy at the first trimester (2.8 ± 0.8 vs. 2.1 ± 0.5; *p* = 0.06).

### Correlations between TTV and available variables

Considering only the first trimester of pregnancy, no difference in the median age among TTV-positive (30 years) and TTV-negative (32 years) women was found (*p* = 0.36).

Neither the presence of TTV (*p* = 0.65) nor TTV loads were associated with a condition of BV (BV: 2.7 ± 0.7; *I*: 2.8 ± 0.9; *H*: 3.2 ± 0.9; *p* = 0.10). Conversely, the presence of TTV was significantly more common in women with a higher vaginal WBC count (37.7 vs. 57.7%; *p* = 0.02). In line with these findings, the levels of IL-6 (median, range: 0.81, 0.01–57.8 vs. 0.41, 0.0–31.2 pg/mL; *p* = 0.02), as well as IL-8 (1,901, 34–35,783 vs. 652, 11.3–43,248; *p* = 0.03) were significantly higher in TTV-positive vaginal samples. Moreover, we observed a trend in the correlation between TTV loads and IL-8 levels (*R* = 0.19; *p* = 0.09). The detection of vaginal *Candida* spp. was not significantly associated with the vaginal presence of the virus nor with higher TTV loads (*p* > 0.5).

On the contrary, several correlations were observed between TTV presence/loads and the levels of particular vaginal metabolites. In relation to this, it is worth mentioning that a total of 63 metabolites were detected in the vaginal cell-free supernatants, mainly belonging to the groups of SCFAs, organic acids, amino acids, and biogenic amines (Supplementary Table S1).

In particular, TTV-positive samples were characterized by higher levels of propionate (median, range: 0.01, 0.001–0.36 vs. 0.007, 0.001–0.23 mM; *p* = 0.001) and cadaverine (0.008, 0.002–0.05 vs. 0.006, 0.001–0.06 mM; *p* = 0.006), compared to TTV-negative ones.

Moreover, TTV titer was positively correlated with the levels of 4-hydroxyphenyllactate (*p* < 0.0001), isoleucine (*p* = 0.01) and phenylalanine (*p* = 0.04). Vaginal molecules showing a negative correlation with TTV loads included benzoate (*p* = 0.008), inosine (*p* = 0.002), and creatine (*p* = 0.004) (the full list is displayed in Table 1).

**Table 1:** List of the vaginal molecules, whose concentration was found related to TTV loads.

	**Spearman *r***	***p*** **-value**
Xanthine	−0.276	0.01
Benzoate	−0.298	0.008
Phenylalanine	0.231	0.04
Tyramine	−0.259	0.02
4-Hydroxyphenyllactate	0.508	<0.0001
Inosine	−0.336	0.002
Uridine	−0.2285	0.04
Uracil	−0.265	0.02
Methanol	−0.256	0.02
Ethanolamine	−0.261	0.02
Creatinine	−0.2780	0.01
Creatine	−0.320	0.004
Asparagine	−0.288	0.01
TMA	−0.301	0.007
2,3-Butanediol	−0.285	0.01
Propionate	−0.366	0.001
Isoleucine	0.265	0.02

Statistical significance was searched by Spearman correlation coefficient.

### Correlation between TTV and vaginal microbiome profiling

**Figure 1:**
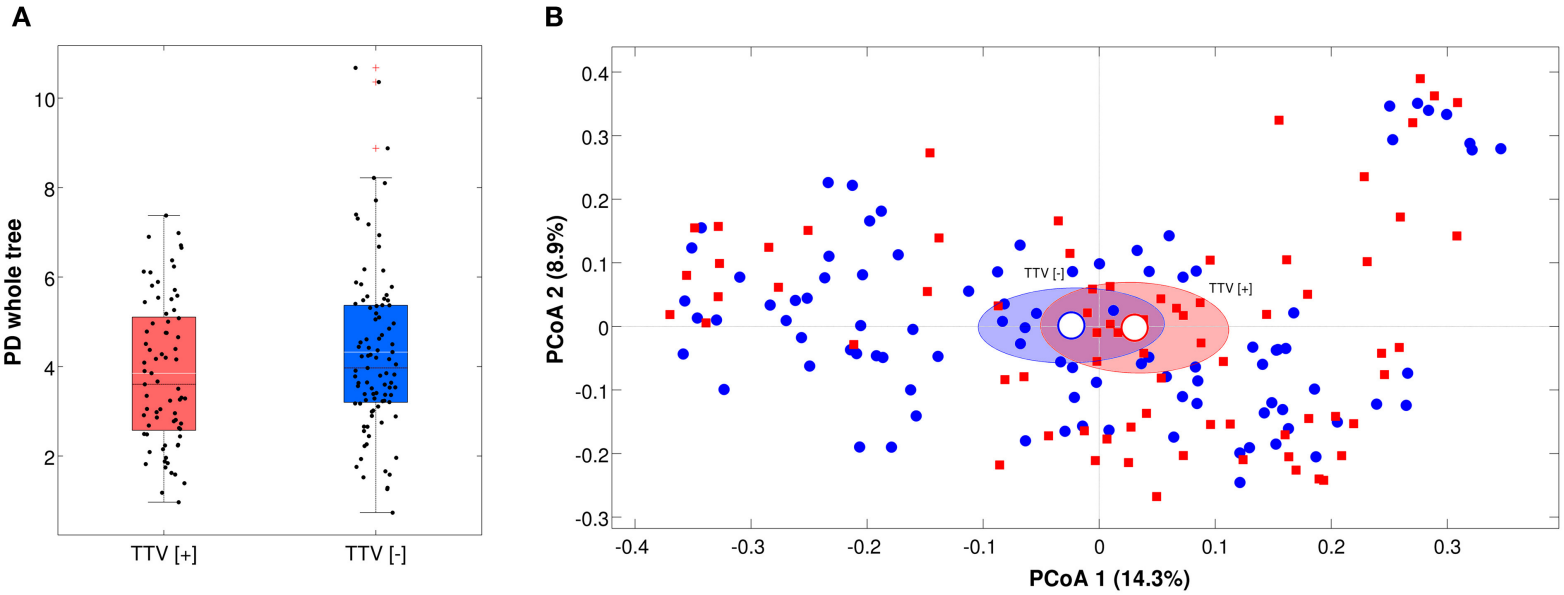
**(A)** Boxplot of the alpha-diversity according to Faith's phylogenetic diversity metric, grouped by TTV presence or absence. Each point represents a sample; median of the distributions are in black, whereas means are in white; **(B)** Principal Coordinate Analysis (PCoA) of the beta-diversity values according to unweighted Unifrac distances. Each point represents a sample; data points are colored according to the presence or absence of TTV; ellipses represent the 95% SEM-based confidence intervals; the first and the second coordinates are represented.

For microbiota analysis, only samples with a number of reads >5,000 (*n* = 175) were considered, in order to have a reliable picture of the microbial composition. TTV-positive and negative samples showed no statistical difference (*p* > 0.05) on both biodiversity (alpha-diversity) or microbial composition (beta-diversity) for all the metrics considered (Figure 1).

Moreover, the analysis of the bacterial relative abundances did not reveal any major changes in the bacterial groups between TTV+ and TTV- samples. Nevertheless, we noticed significant differences in two low-abundant taxa (average relative abundance <1%), such as a higher abundance of *Sneathia* (0.92 vs. 0.29%; *p* = 0.04) and *Shuttleworthia* (0.89 vs. <0.01%; *p* = 0.0009) in TTV-positive samples.

The point-biserial correlation confirmed the significant positive correlations between TTV presence and the abundance of *Sneathia* (*R* = 0.123) and *Shuttleworthia* (*R* = 0.125). Spearman analysis showed a significant correlation between TTV loads and the abundance of *Sneathia* (*R* = 0.166) and *Shuttleworthia* (*R* = 0.313).

Although not statistically significant, TTV-positive samples were characterized by a decrease of *L. crispatus* (32 vs. 41%) and *L. jensenii* (7 vs. 10%), as well as by an increase of *L. iners* (25 vs. 15%), compared to TTV-negative ones.

In this context, it is worth mentioning that we found a negative correlation between the levels of pro-inflammatory cytokines and both *L. crispatus* (*R* = −0.354 and *R* = −0.277 for IL-6 and IL-8, respectively) and *L. jensenii* (*R* = −0.309 and *R* = −0.171 for IL-6 and IL-8, respectively).

## Discussion

The presence and role of TTV in pregnant women is still only scarcely available, so this study aimed to provide new insights into the dynamics of TTV in the vaginal ecosystem during pregnancy. We explored TTV presence and loads in a cohort of white pregnant women at different gestational stages and we assessed its correlation with the vaginal bacterial composition, with the vaginal metabolic profiles and with the vaginal concentration of two pro-inflammatory cytokines.

In line with previous findings (Tozetto-Mendoza et al., 2022), we observed that TTV is quite common in women with normal pregnancy outcomes, with a prevalence ranging from 53% at the first trimester to 36% at the third. This is not surprising if we consider that TTV has been identified both in peripheral blood and in cervical/vaginal fluids (Maggi and Bendinelli, 2010; Chen et al., 2011; Tozetto-Mendoza et al., 2020).

No difference in TTV prevalence and loads was observed between women with normal pregnancies and miscarriages. Even though further studies including a larger cohort of women are needed for a better comprehension of TTV role during pregnancy, these results seem to indicate that TTV does not have clinical outcome consequences.

Interestingly, TTV presence was positively related to the number of vaginal WBC, as well as to higher concentrations of vaginal proinflammatory cytokines (i.e., IL-6 and IL-8). This result is not surprising if we consider that TTV has been recognized as a predictor of local immune status (Focosi et al., 2016). It has been speculated that, in the vaginal ecosystem, TTV loads are related to the presence of activated lymphoid cells, being the vaginal TTV an additional indicator of the local “immune” status in pregnant women (Brundin et al., 2020; Tozetto-Mendoza et al., 2022).

Other interesting data emerged when TTV presence and loads were related to the vaginal bacterial composition. The most significant results included (i) the association between TTV and higher levels of *Sneathia* and *Shuttleworthia*, (ii) a trend toward a decrease of *L. crispatus* and *L. jensenii*, as well as an increase of *L. iners* in TTV-positive samples.

In this context, it is worth underlining that a significant negative correlation between *L. crispatus* and *L. jensenii* and both IL-6 and IL-8 was observed.

Since TTV replication preferentially occurs in activated lymphoid cells (Brundin et al., 2020), and immune system activation is at its lowest level in case of a *L*. *crispatus*-dominated vaginal microbiome (Witkin and Linhares, 2017), we can speculate that the absence/decrease of TTV is linked to the reduction of lymphoid cells or their pro-inflammatory molecules when *L*. *crispatus* is predominant. On the contrary, the presence of *L. iners* is associated with a higher expression of genes involved in leukocyte mediated immunity and activation (Mohd Zaki et al., 2022), being potentially associated with higher levels of TTV in the vaginal environment.

The association between TTV and higher levels of *Sneathia* and *Shuttleworthia* probably goes in the same direction. In fact, genital inflammation can be linked to specific BV-related microorganisms, including *Sneathia* (Kaelin et al., 2022). As reported by Lopez-Filloy et al., women with HPV infection are characterized by a significant increase in anaerobes, such as *Sneathia* and *Shuttleworthia*, their presence being in turn associated to a higher level of cervico-vaginal inflammation and a higher risk of BV recurrence (López-Filloy et al., 2022).

In this context, the association between TTV-positive samples and higher concentrations of propionate and cadaverine could reflect these findings. In fact, these two molecules (belonging respectively to SCFAs and biogenic amines) are common markers of vaginal dysbiosis, typically produced by BV-related anaerobes, when a reduction of vaginal lactobacilli is present (Laghi et al., 2021).

In addition, we observed a highly significant correlation between TTV loads and the levels of 4-hydroxyphenyllactate. This metabolite is produced by lactic acid bacteria and exerts both antifungal properties and radical scavenging activities (Mu et al., 2010; Suzuki et al., 2013).

Further studies are needed to understand the exact role and origin of 4-hydroxyphenyllactate and if this molecule can possess antiviral activities against TTV.

We are fully aware of some limitations of this study: (i) for TTV detection, we tested cell pellets after a low centrifugation step instead of supernatants; thus, we mainly detected the presence of the virus inside the host cells, (ii) the association between TTV and specific microbes of the vaginal ecosystem (i.e., *Sneathia* and *Shuttleworthia*) could be a coincidental finding. Additional studies are needed to understand if there is a real biological cooperation or if these microbes are simple bystanders.

In conclusion, in agreement with previous reports (Focosi et al., 2016; Tozetto-Mendoza et al., 2020), we found that TTV is commonly found in the vaginal ecosystem of pregnant women, representing a possible predictor of local immune status. In fact, its detection and loads vary with local vaginal conditions, being more common in presence of higher levels of leukocytes, higher levels of BV-related microbes, and lack of *L. crispatus* dominance.

Future perspectives include the assessment of the clinical role/utility of the vaginal TTV titer in the evaluation of the vaginal immune status, with the goal of opening new diagnostic/prognostic approaches for maternal-fetal health.

## Data availability statement

The datasets presented in this study can be found in online repositories and in the Supplementary material. The names of the repository/repositories and accession number(s) can be found in the article.

## Ethics statement

The studies involving human participants were reviewed and approved by Ethics Committee of Romagna (CEROM) (No. 2032 of 21st February 2018). The patients/participants provided their written informed consent to participate in this study.

## Author contributions

AM, CF, and VS conceived and designed the study. SZ and GP recruited the patients. LL, MD, MP, SM, MS, CCo, CCe, and TC performed the experiments. CF, LL, and MS analyzed the data. AM and VS provided reagents, materials, and analysis tools. CF, AM, and MS wrote the paper. All the authors read, reviewed, and approved the final manuscript.

## Funding

This study was supported by Fondazione del Monte di Bologna e Ravenna (Prot. No. 329bis/2017). The funder had no role in study design, data collection and analysis, decision to publish, or preparation of the manuscript.

## Conflict of interest

The authors declare that the research was conducted in the absence of any commercial or financial relationships that could be construed as a potential conflict of interest.

## Publisher's note

All claims expressed in this article are solely those of the authors and do not necessarily represent those of their affiliated organizations, or those of the publisher, the editors and the reviewers. Any product that may be evaluated in this article, or claim that may be made by its manufacturer, is not guaranteed or endorsed by the publisher.
